# Human *NDE1* splicing and mammalian brain development

**DOI:** 10.1038/srep43504

**Published:** 2017-03-07

**Authors:** S. Mosca, M. Raponi, A. Meneghello, E. Buratti, C. G. Woods, D. Baralle

**Affiliations:** 1 Human Development and Health, Faculty of Medicine, University of Southampton, UK; 2 Department of Pathology, University of Cambridge, Cambridge, UK; 3 International Centre of Genetic Engineering and Biotechnology, Trieste, Italy; 4 Cambridge Institute of Medical Research, University of Cambridge, Cambridge, UK

## Abstract

Exploring genetic and molecular differences between humans and other close species may be the key to explain the uniqueness of our brain and the selective pressures under which it evolves. Recent discoveries unveiled the involvement of Nuclear distribution factor E-homolog 1 (*NDE1*) in human cerebral cortical neurogenesis and suggested a role in brain evolution; however the evolutionary changes involved have not been investigated. *NDE1* has a different gene structure in human and mouse resulting in the production of diverse splicing isoforms. In particular, mouse uses the terminal exon 8 T, while Human uses terminal exon 9, which is absent in rodents. Through chimeric minigenes splicing assay we investigated the unique elements regulating *NDE1* terminal exon choice. We found that selection of the terminal exon is regulated in a cell dependent manner and relies on gain/loss of splicing regulatory sequences across the exons. Our results show how evolutionary changes in cis as well as *trans* acting signals have played a fundamental role in determining *NDE1* species specific splicing isoforms supporting the notion that alternative splicing plays a central role in human genome evolution, and possibly human cognitive predominance.

Alternative splicing is a complex mechanism used to regulate gene expression. This process allows different mature mRNAs to be produced from the same gene. The resulting diversity in amino acid sequence/content of the translated proteins isoforms may provide different biological functions^1^. Alternative splicing is regulated during the transcription and premRNA maturation, with splicing factors (regulatory proteins) interacting with *cis*-acting sequences in the pre-mRNA^2^,^3^. Changes in splicing regulatory sequences and proteins have strongly contributed to genome diversity by shaping alternative splicing signatures^4^,^5^,^6^,^7^,^8^. In this study we investigate splicing differences in the gene *nudE nuclear distribution gene E homolog 1 (NDE1*). NDE1 produces a protein vital to normal brain development. The gene is found on chromosome 16 and plays essential roles in microtubule organization, mitosis and within the central nervous system. Whilst this gene is crucial for cerebral cortex development it is also required for centrosome duplication, formation and function of the mitotic spindle^9^. The major NDE1 transcript has nine exons and the corresponding protein has a predicted self-association domain, a LIS1 interaction domain and a NUDE_C domain (c-terminal conserved region)^10^. In human pathology, NDE1 was identified via its interaction with Lissencephaly 1 (*LIS1*). The protein^11^ has a role in brain size and cortical lamination and possibly schizophrenia (via an interaction with the DISC1 protein)^12^,^13^. The splicing pattern of *NDE1* is known to differ in human and mouse^14^, with mouse using terminal exon “8 T”, while human uses terminal exon 9, usually missing exon 8 T; generating different splicing products and different Nde1 proteins at the C-terminal domain. Interestingly, exon 8 T in human is included in the mature transcript thanks to the use of an alternative 5′ss and is joined to exon 9. This minor human transcript uses exon 8 T as a terminal coding exon due to the presence of an in frame stop codon. As a result, there appears to have been a change through mammal evolution that must have led to a decreasing 8 T use and increasing 9 use (that is humans almost obligatory) (Fig. 1A,B). An interesting observation is that deletion of *NDE1* produces mice with small brains but humans with homozygous loss of this gene have a severe phenotype with severe microcephaly and mental retardation)^14^. Part of this significant clinical difference may be explained by the differences in the splicing process between the two models.

In this study, we characterize the splicing pattern in mouse and human models of *NDE1*, to understand how this different terminal exon usage has evolved in these two organisms. Using primary brain samples and minigene approaches, our results show that evolutionary changes in *cis* as well as *trans* acting elements are essential determinants of *NDE1* terminal exon choice.

## Results

### Characterization of *NDE1* splicing pattern: differences between human and mouse

Endogenous *NDE1* expression was tested in several human and mouse tissues to assess eventual differences in NDE1 splicing products. Human NDE1 expression was analysed by examining fetal human tissues (kindly prepared by Professor *D. Wilson, Southampton*), and in HeLa, SK-N-SK and Hek-293 cell lines. With endogenous NDE1 primers (Materials and Methods, 2.4) we obtained three bands for each sample (Fig. 2A). Two of the three products observed were as expected: a higher band corresponding to the *full-* length splicing product of *NDE1* including exon 7, exon 8, exon 8 T and exon 9 (Fig. 2A, lanes 1 to 6 and 8). A more intense band, corresponding to exon 8 T skipping was present in all samples (Fig. 2A, lanes 1 to 8). Interestingly a third lower band was also observed in all samples (Fig. 2A, lanes 1 to 8), representing a new splice isoform, not previously reported, that is formed following exclusion of both exon 8 and exon 8 T in humans. We called this isoform “Delta8” (∆8). The identity of all these bands was confirmed by direct sequencing, quantification is reported below each lane. In parallel, we analysed endogenous mouse *NDE1* expression in several mouse tissues (kindly prepared by *Dr Eve and Dieter Riethmacher*) and cell lines, Neuro2a, and Mouse fibroblast cell lines. Using mouse endogenous *NDE1* primers (Materials and Methods, 2.4) we obtained two bands: the higher band originated from use of an alternative 3′ splice site in exon 8 T (Fig. 2B, lanes 1 to 8); and a more intense band corresponding to the main splicing isoform that includes the joining together of exons 7, 8, and 8 T (Fig. 2B, lanes 1 to 8). In mouse, exon 9 is never used. The identity of these mouse RT-PCR bands were confirmed by sequencing (data not shown). Mouse uses a 3′UTR following 8 T while in human the inclusion of exon 8 T is triggered by a 5′ss 32 bp downstream the stop codon.

To extend our knowledge of the relationship between the two models, we transfected SK-NSH, Hek-293, HeLa, Mouse Fibroblast and Neuro2a cell lines with human and mouse minigenes that carried these sequences (see Materials and Methods, 2.1; 2.3) (Fig. 3A, lanes 1-5). The splicing pattern observed in the minigenes was similar to endogenous expression although some differences in band intensities were observed, as confirmed by quantification. In particular, cells with human minigenes show three bands with different intensities that, when sequenced, were confirmed to represent the following splicing events: full transcript (7-8-8T-9), skipping of exon 8 T (7-8-9) and skipping of exon 8, ∆8 isoform (7–9) (Fig. 3A, lanes 1–5). Similarly, the two bands observed from the mouse minigene were shown to correspond with isoform including exons 7, 8, and 8 T but not exon 9. As observed in the endogenous gene, the difference in size in the two products is due to the use of an alternative 3′ splice site in exon 8 T (Fig. 3B, lanes 1–5). Interestingly the percentage of exon 8 T exclusion for the human minigene was much higher in mouse (Mouse Fibroblast and Neuro2a, 79% and 65% respectively, Fig. 3A lanes 4–5) compared to human cell lines (SH-N-SH, Hek-293 and HeLa, 40%, 37%, and 39% respectively, Fig. 3A lanes 1–3).

To understand the evolution in usage of the terminal exon 8 T and 9 we compared the sequence of these exons between human, mouse and Gallus and analysed the mechanism of modulation of the splicing process. The nucleotide differences between these regions are highlighted in (Fig. 4). Differentiated fibroblast cell lines derived from Gallus (dt40 cell line) kindly prepared by *Marco Baralle, (ICGEB, Trieste*) show the same NDE1 splicing pattern as mouse fibroblast (data not shown). As shown in Fig. 4, this comparison allowed identification of several nucleotide sequence differences that could be potentially responsible for the different splicing patterns observed in the various human and mouse tissues/cell lines.

### Terminal exon choice is modulated by cis-acting sequences

In order to address the potential significance of these nucleotide differences, several nucleotides were substituted in the human NUDE1 minigene with the corresponding mouse sequence by site-directed mutagenesis (favouring common sequences between gallus and mouse) (Fig. 4). In this way one minigene for each mutation was generated which was transiently transfected into SK-N-SH cell lines for splicing analyses (Fig. 5A). Nucleotide substitutions on human exon 9 in minigenes M9c (G > C), M9g (A > G), M9gc (A > G and A > C) produced a weak effect on exon 9 splicing by favouring the production of the 8 TplusEx9 isoform (Fig. 5B, lanes 4–6). The strongest effect was observed with M9g with increased inclusion of exon 8 T (Fig. 5B, lane 5, 60% inclusion). We also analysed the effect of the chimeric minigene 8th9m in which the entire exon 9 was replaced with mouse exon 9. In this case, we see a stronger effect on exon 8 T inclusion (85%) favouring the production of the full isoform including all exons (Fig. 5B lane 2). The effects of nucleotide substitutions on exon 8 T in human minigenes that carried the following substitution was also analysed: M1 (G > A and G > A; Figs 4B and 5A) and M3 (C > G, C > G, T > C, G > A, T > G, T > A, T > C, G > A, C > G; Figs 4B and 5A). As shown in Fig. 5C, lanes 2–4, the minigenes carrying substitutions M1 and M3 decrease the inclusion of exon 8 T to 37% and 26% respectively, with the exception of M2 (G > A and A > G) that increases inclusion to 57%. The splice factors tested were chosen by looking at the nucleotide substitutions in exon 8 T, and observing which consensus sequences were affected (Fig. 4B), as predicted using SpliceAid^15^.

### Changes in splicing factor expression mediate *NDE1* alternative splicing

In order to investigate the role of splicing factors in the human *NDE1* splicing, we focused on polypyrimidine tract-binding proteins (PTB), neural PTB (nPTB) and heterogeneous nuclear ribonucleoprotein H (hnRNP H), as these factors have consensus sequences in human exon 8 T (Fig. 4B) that are disrupted in the generated mutant M1 (hnRNP H) and M3 (PTB). We performed knockdown experiments of these factors in SK-N-SH cell lines transfected with the human or mutated M1, M3 minigenes.

Western blot was performed to confirm knock down (Supplementary Figure 1A). Using ImageJ software (http://imagej.nih.gov/ij/), the amplified cDNA bands from the splicing assay were measured for calculation of percentage skipping of exon 8 T in each sample. We observed no statistically significant difference in the splicing pattern of *NDE1* between hnRNP H knockdown and control siRNA (Supplementary Figure 1B).

We the analysed the contribution of PTB and its neuronal form nPTB to NDE1 terminal exon splicing. The human NDE1 minigene was transfected in the following circumstances: 1) with both PTB and nPTB knockdown (Fig. 6A, lane 2); 2) PTB knockdown plus overexpression of nPTB (Fig. 6A, lane 3) 3) nPTB overexpression (Fig. 6A, lane 4) and compared to 4) siRNA control with the human NDE1 minigene transfection (Fig. 6A, lane 1).

Unlike hnRNP H, these experiments showed important changes between control conditions and the siRNA assay for PTB and nPTB (Fig.6 A,B). When PTB and nPTB were knocked down, we observed increased skipping of exon 8 T (Fig. 6A, lane 2, from 43% to 24%). In conditions where nPTB is overexpressed we see similar results to the control, showing that nPTB is enough to rescue the knockdown effect (Fig. 6A, lanes 3 and 4). To analyse whether this PTB/nPTB effect was dependent on the regulatory sequence predicted in exon 8 T, we repeated the same experiment analysing the differences between control conditions and PTB siRNA following transfection of the mutant minigene M3 (which does not contain the consensus sequence in exon 8 T for PTB or nPTB). The results reported in Fig. 6B, lane 2 show very little no changes in the splicing pattern of the mutant M3 minigene following knockdown of PTB/nPTB confirming that the mutated region in the M3 minigene is a binding site for PTB.

## Discussion

*Nde1* has recently been shown to be critical for cerebral cortex development and mutations in the gene are involved in severe disorders of microcephaly (reduction of brain size, but particularly of cerebral cortex size) and lissencephaly^10^,^13^,^14^. Interestingly a more severe phenotype was seen in human microcephaly compared with mice with the same gene fault^14^. We postulate that this can be in part explained by differences in alternative splicing processes between the two models; given that mouse and human *NDE1* produce different terminal exons.

Several studies have described how alternative gene splicing across genomes can lead to loss or gain of particular traits by subtle alteration of genes function^16^,^17^,^18^,^19^,^20^,^21^. Of relevance, Lin *et al*. reported the existence of human specific splicing patterns in the brain; that such patterns affect protein domains, and are greatly influenced by changes in *cis*regulatory sequences^5^. Another study indicated that changes in conserved cis- regulatory elements of splicing are responsible of the majority of differences in alternative splicing in human and mouse and suggest that also changes in splicing factors acting in trans contributes to the evolutionary landscape of alternative splicing^22^.

In this work, we analysed the difference in *NDE1* splicing patterns in human and mouse, focusing on terminal exon choice and the regulatory elements involved. Analysing cell lines and mouse tissues, we observed that exon 8 T is always the last exon and exon 9 is never included in mouse. Conversely, analysing human fetal tissue and cell lines we show that human *NDE1* produces a main splicing isoform excluding exon 8 T, with exon 9 as the terminal coding exon plus a minor isoform including both exon 8 T and 9 (exon 8 T would be the terminal coding exon). This is in accordance with previously reported work in adult human tissues^23^ (note that in Bradshaw *et al*. exon8T = 8b, exon9 = 8a). Interestingly, we also saw an additional splicing event corresponding to skipping of exon 8 and 8 T together not previously reported. Exon 8 is 152 nucleotides long. Its exclusion from the final human *NDE1* trancript causes frameshift and a new stop codon used in exon 9. The putative protein differs by 80 aminocids at the c-terminal compared to the main isoform. This appears in all our experiments (both endogenous and human minigene analysis) and better defines the complexity of the alternative splicing process of *NDE1*.

Therefore at least 3 different c-termini are produced in human *NDE1* proteins, while only one is produced in mouse, corresponding to the minor human isoform including exon 8 T. The 3 possible c-termini in human *NDE1* are: GLDTSCRWLSKSTTRSSSSC (main isoform); VGHELPLVVQINNQVVQLLLKPVLGLFQFIISGRLLLVLLGEVLDLLLEPLLPLFQSFH GAGKVLQLLLRVGELQGQRPG (isoform skipping exon 8); GLGKRLEFGKPPSHMSSSPLPSAQGVVKMLL (isoform including exon 8 T).

This is highly important from an evolutionary point of view since crucial amino acids for *NDE1* function (interaction with other protein at the centromere) have been described in the c-terminus of the main human isoform (for review see^24^). These include a palmitoylation site (amino acid C274), and 7 phosphorylation sites (Y279, S282, S291, S306, S307, T308, S309) corresponding to exon 8.

Although particular active amino acid sites have not been reported for *NDE1* in correspondence to exon 9, an important role of this exon in *NDE1* function should not be excluded. For instance, *NDE1* exon 9 may contain important regulatory sequences for RNA transport and/or stability that in turn might regulate the expression of *NDE1* at protein level.

The c-terminal domain of *NDE1* produces different intra and inter species *NDE1* isoforms and we have established the elements regulating selection of the terminal coding exon in humans. We identified these elements using chimeric minigenes for splicing assay and comparing alternative splicing regulation between mouse and human cell lines. Intriguingly, transfection of the human minigene resulted in a different splicing pattern when transfected in mouse cell lines compared to human cell lines, suggesting that expression level of transacting factors also plays an important role in this process. For example, exon 8 T was almost completely skipped from the human minigene pre-mRNA in mouse cell lines. This suggests the presence of different regulatory trans acting factors of splicing between human and mouse; or a different functionality of those factors between the two species.

With this pattern the role of *cis* and *trans* elements in the regulation of the *NDE1* alternative splicing process are likely to be important. To investigate the role of *cis* elements we substituted human nucleotides in exon 8 T and exon 9 with mouse mismatches and we investigated changes in the splicing process. Replacement of human exon 9 with the mouse exonic sequence in the minigene human exon 8 T/mouse exon 9 resulted in inclusion of human exon 8 T (Fig. 6A). However, single nucleotide substitutions were only in part able to recapitulate this effect, suggesting the presence of regulatory *cis* acting elements of splicing regulation all along exon 9, which act cooperatively.

We also identified an important silencer regulatory region in human exon 8 T, which when mutated (M2, Figs 4 and 5) results in increased inclusion of exon 8 T. Conversely, another two regions in human exon 8 T seem to act as enhancers of this exons’ splicing; and these regions mutated (M1 and M3) result in increased skipping of exon 8 T.

We subsequently focussed on PTB and nPTB since these important regulatory proteins of splicing are involved in brain development^25^ and were predicted by SpliceAid to bind human *NDE1* exon 8 T (the enhancer region mutated in the M3 minigene) but not mouse.

Knock down of these splicing regulatory proteins had a strong effect on exon 8 T splicing of the human minigene demonstrating their role in the regulation of NDE1 processing. On the other hand, knockdown of PTB/nPTB had no effect on the minigene with the mutated region M3, confirming a central role of this sequence in the regulation of exon 8 T inclusion through binding of PTB. These results also confirm the role of PTB as an enhancer of *NDE1* exon 8 T splicing. This is an unusual mechanism of splicing regulation by PTB which normally acts as a silencer of splicing, here essentially acting as an enhancer of exon inclusion through binding of elements in the downstream intron rather than within the exon^26^. Moreover, this, represents a mechanism of splicing regulation through an enhancer sequence which is unusually distant from the splice sites (48 nucleotides from the 3′ss and 78 nucleotides from the 5′ss). Overall this finding underlines the importance of performing comparative genomic analysis to uncover unusual splicing regulatory mechanism which would be otherwise underestimated.

In conclusion, we provide an overview of the *NDE1* splicing process in human and mouse models: model mammals with particularly large and small cerebral cortices respectively. We describe a new isoform excluding exons 8 and 8 T, that PTB/nPTB are important splice factors functioning as distant enhancers, and that this has changed during species evolution as has cerebral cortex size. Future work would include testing other splicing regulatory elements responsible for exon 8 exclusion and to investigate the function of different *NDE1* c-terminal domains.

## Methods

### Minigene construction

**Figure 1:**
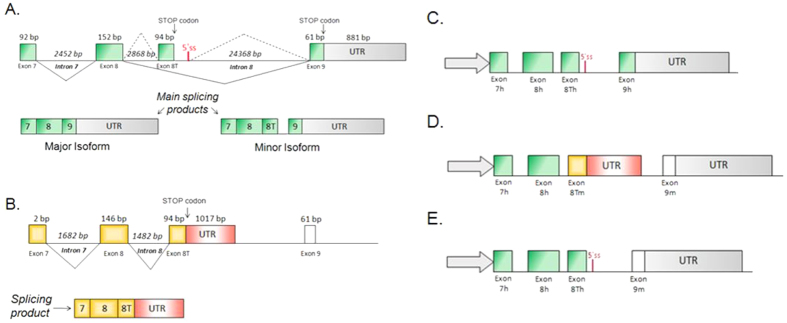
Alternative splicing scheme in human (green/gray) and mouse (orange/red) models and schematic representation of minigene constructs used in this study. Grey arrows represent pCDNA3 vector where the partial NDE1 genomic sequence was cloned under the control of the CMV promoter. (**A**) Terminal human NDE1 structure. The main UTR region is after exon 9. It is interesting to note that after exon 8 T there is a 5′splice site, this produces the minor isoform including exon 8 T with its own stop codon, part of intron 8 and exon 9. Continuous lines represent major splicing events, dotted lines represent alternative splicing events with exon 8 T inclusion. (**B**) Terminal mouse NDE1 structure. The gene uses exon 8 T as its terminal exon; the splicing product excludes exon 9; continuous lines represent the constitutive splicing event. (**C**) The human minigene construct consisting of human exon 7, exon 8, exon 8 T and exon 9, including partial intron flanking sequence. The 3′ UTR belongs to pCDNA3+. (**D**) The mouse minigene construct consisting of human exon 7, exon 8, mouse exon 8 T and mouse exon 9; including partial intron flanking sequences. The 3′ UTR belongs to pCDNA3+. (**E**)The chimeric minigene called 8Th9m is a human minigene with the human exon 9 replaced by the mouse exon 9. The 3′ UTR belongs to pCDNA3+.

**Figure 2:**
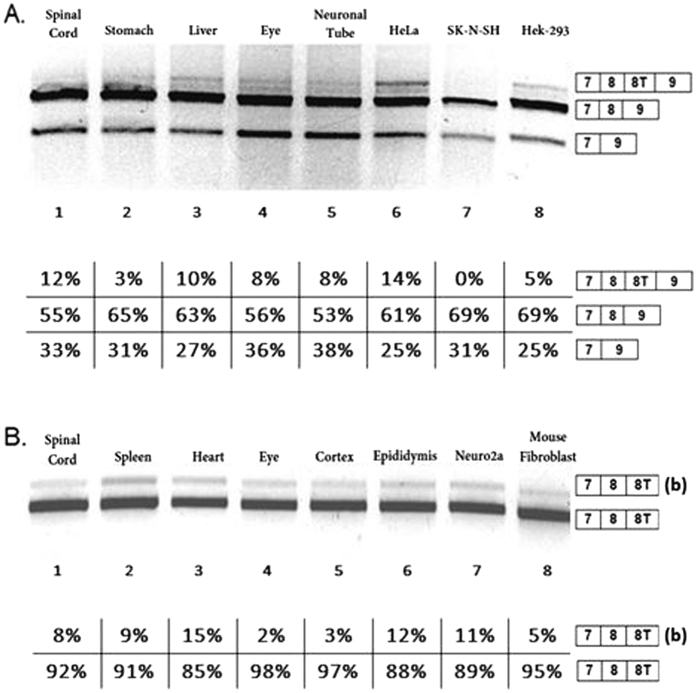
Analysis of endogenous NDE1 splicing. All gels were run under the same experimental conditions and the experiments were repeated at least 3 times. (**A**) Endogenous NDE1 splicing pattern in human tissues (from the same foetus) and human cell line (HeLa, SK-N-SK, Hek-293)s; The oligonucleotides used to amplify the endogenous gene were positioned in exon 7 (forward oligo) and 9 (reverse oligo). (**B**) Endogenous NDE1 splicing pattern in mouse tissues (from the same mouse) and mouse cell lines (Neuro2a and mouse fibroblast). The percentage of exon 8 T inclusion is 100% in all samples. The higher band indicated with (b) is originated from the use of an alternative 3′ splice site upstream the canonical 3′ss of exon 8 T. The oligonucleotides used to amplify the endogenous genes were positioned in exon 7 (forward oligo), 8 T (reverse oligo) and pseudo exon 9 (reverse oligo) in mouse.

**Figure 3:**
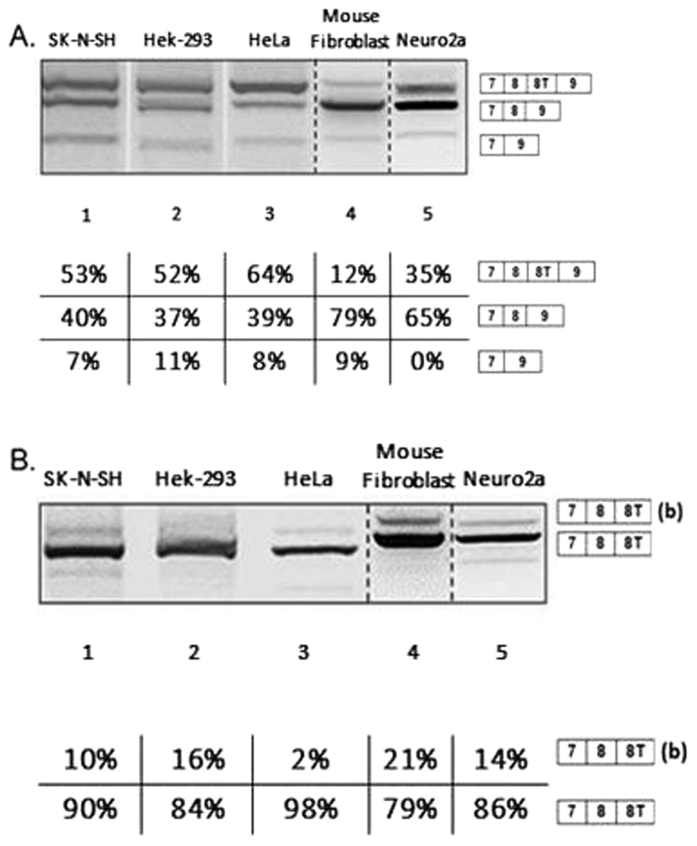
All gels were run under the same experimental conditions and the experiments were repeated at least 3 times. Dotted lines indicate cropping within the same gels. (**A**) NDE1 splicing pattern in cell lines transfected with human NDE1 minigene. (**B**) NDE1 splicing pattern in cell lines transfected with mouse NDE1 minigene. The higher band indicated with (b) is originated from the use of an alternative 3′ splice site upstream the canonical 3′ss of exon 8 T. The faint lower band was not characterised due to the inability to sequence it.

**Figure 4:**
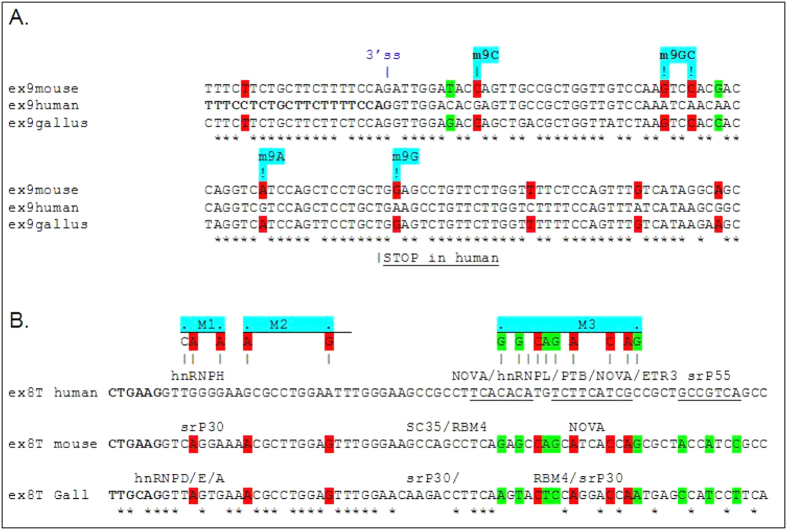
Exon 9 and 8 alignment. Alignment was performed using Ensemble (http://www.ensembl.org/index.html). In red are highlighted the common mismatches between human and mouse sequence; in green are highlighted the mismatches between all three organisms. The star (*) indicates conserved nucleotides. The sequence changes created separately in the human minigene for transforming it to a more mouse-like genotype are highlighted in blue. (**A**) Alignment of exon 9. The 3′ss, upstream human exon 9, is shown in bold. The human stop codon is underlined. (**B**) Alignment of exon 8 T. Part of the 3′ss upstream exon 8 T is shown in bold characters. Splicing regulatory proteins predicted with SpliceAid to bind exon 8 T are indicated.

**Figure 5:**
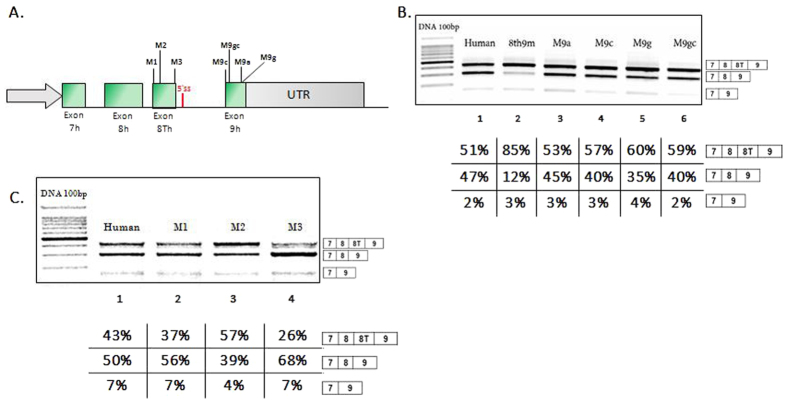
NDE1 Splicing pattern in SK-N-SH cells transfected with minigenes for each mismatch analysed. All gels were run under the same experimental conditions and the experiments were repeated at least 3 times. (**A**) Scheme of minigenes with nucleotide substitutions in exon 8 T and 9. (**B**) Analysis of exon 9 mutants and of human minigene with mouse exon 9. (**C**) Analysis of exon 8 T mutants.

**Figure 6:**
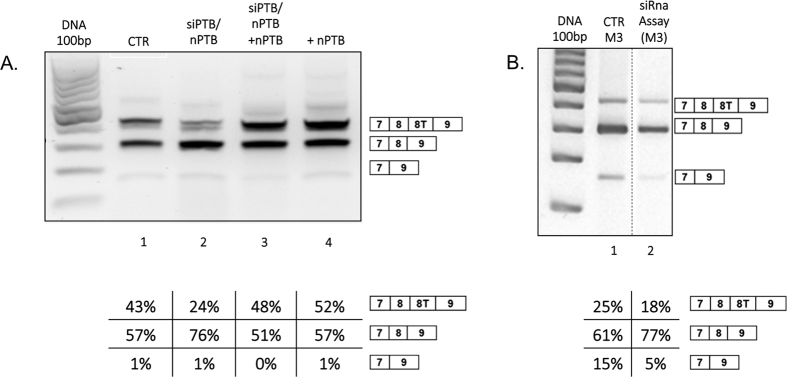
Analysis of the effect of PTB and nPTB on NUDE1 and M3 minigenes splicing. Gels were run under the same experimental conditions and the experiments were repeated at least 3 times. Dotted lines indicate cropping within the same gels. (**A**) NDE1 splicing pattern in SK-N-SH cells transfected with human NDE1 minigene and (1) luciferase siRNA control; (2) siRNA for PTB and nPTB; (3) siRNA for PTB and nPTB, but with overexpression of nPTB (siRNA resistant^27^); (4) human NDE1 control with nPTB overexpression. (**B**) Chart showing band intensity percentage of 8 T skipping, relative to panel A; (**C**) NDE1 splicing pattern in SK-N-SH cells transfected with M3 minigene. (1) Control luciferase siRNA and transfection of tM3 minigene;(2) siRNA for PTB and nPTB with human NDE1 minigene transfection. (**D**) Chart of band intensity percentage and skipping of exon 8 T, relative to panel C.

The minigenes containing different combinations of mouse or human exons are shown in Fig. 1C,D,E. They consist of four exons (including part of their flanking introns). These were cloned in a modified version of the pCDNA3(+) vector (Invitrogen) under CMV promoter control. 4 inserts were amplified and cloned separately with specific primers and restriction enzymes. Restriction enzymes used for cloning were HindIII/KpnI for exon 7 (insert 1); KpnI/BAMHI for exon 8 (insert 2); BAMHI/Xho for exon 8 T (insert 3) and Xho/Xba for exon 9 (insert 4). Digested PCR products were purified by gel electrophoresis and ligated into a linearized pCDNA3 vector. Ligation reactions were incubated overnight at room temperature and added to competent Escherichia coli strain DH5a for transformation. Bacterial colonies were grown in LB broth containing ampicillin at a final concentration of 50 mg/ml. Plasmid DNA was extracted using Miniprep kit of Qiagen and sent for sequencing.

### Cell Culture

Several mammalian cell lines were used for transfection experiments including SK-N-SK (human neuroblastoma cell line), Hek-293 (Human Embryonic Kidney 293 cells), HeLa (human cervical cancer immortal cell line), Neuro2A (murine neuroblastoma cell line) and Mouse fibroblast cell line. Mouse project licence, PPL30/2692. Fetal tissues were obtained with informed consent and according to the protocol ethically approved by Southampton and South West Hants LREC. Cells were grown and maintained in Petri dishes or 75 mm^2^ flasks at 37 °C with 5% CO_2_ in Gibco^®^ DMEM Dulbecco’s Modified Eagle Medium (*Life Technologies*) enriched with 10% Gibco^®^ FBS (*Fetal Bovine Serum; Life Technologies*). Antibiotics (*100 μg/ml Penicillin, Streptomycin; Sigma Aldrich*) were added when required. Once the cultures reached the appropriate plating density, they were split and medium was replaced. The desired concentration of cells to be plated was determined by counting the cultured cells with the Neubauer Chamber^2^ and dispensing the correct volume of suspension into the well. Cell cultures were preserved by freezing and storing them at −80 °C. Cells were trypsinized, pelleted and resuspended in 1.5 ml of freezing medium (90% FBS and 10% DMSO).

### siRNA, transfection and PCR assays

Mouse minigene and Human minigene were transfected into Mouse fibroblasts and Neuro2a following standard protocols with a 3:1 FuGENE^®^ 6 Transfection Reagent:DNA ratio (*Promega*). For silencing, 20 nM siRNAs for PTB and nPTB^27^ and 20 nM siRNA for hnRNP H (*Sigma*) were transfected separately into 28 × 10^3^ SK-N-SH cell line with the INTERFERin^®^ reagent (*Polyplus transfection*). Cells were grown overnight and fresh complete medium was added to repeat the siRNA assay. Cells were grown for additional 48 h for siPTB and sinPTB assays and 24 h for sihnRNP H assay. Total RNA was extracted using an RNeasy mini kit (*Qiagen, Valencia, CA, USA*) according to the manufacturer’s instructions. Total RNA (1 μg) was reversetranscribed using random hexamer primers (*Promega*) and cDNA was then amplified by PCR using the FastStart Taq DNA Polymerase (ROCHE) in a total volume of 50 μL with primers specifically designed to amplify processed transcripts derived from the minigene (95 °C for 4 minutes, followed by 95 °C for 30 seconds, 54 °C for 30 seconds, 72 °C for 45 seconds, and final extension at 72 °C for 7 minutes). Each transfection experiment was performed at least in triplicate.

### Primers

**Human:**

nudHex7 F: 5′-ATATCAGCCCTCAACATTGT,

9endR: 5′-AGACCAAGAACAGGCTTCA

NewT7 F: 5′-GACTCACTATAGGGAGACCCA,

Hex9Rev: 5′-TTGGACAACCAGCGGCAACT

**Mouse:**

NewT7 F: 5′-GACTCACTATAGGGAGACCCA,

NewM8T R: 5′-TGGCTCTTATTTCTCAAACTAC,

Hex9Rev: 5′-TTGGACAACCAGCGGCAACT.

### Western blot

siRNA assays samples were electroblotted onto a Hybond-C Extra membrane (*Amersham, Chalfont St Giles, UK*), and antibody recognition was performed using in-house or commercial antibodies. A blocking solution was added to cover the membrane: the buffer contained 3–5% lyophilised milk for alimentary use, 1X PBS and 0.1% Tween20. The membrane was incubated for at least one hour and washed again with 1X PBS. The membrane was consequently incubated with the specific primary antibody, diluted in 1X PBS + 0.1% Tween20 and 3% BSA, for 1–2 hours. The membrane was washed with 1X PBS three times, and incubated with the appropriate secondary antibodies for 1–2 hours and washed again. Protein bands were detected using an enhanced chemiluminescence kit (*Pierce, Rockford, IL, USA*) according to the manufacturer’s instructions. The following antibodies were used in this study: Polyclonal Anti-PTB antibody produced in rabbit (*in house from ICGEB, Trieste, Italy*); dilution: 1:1000. Polyclonal Anti-hnRNP H antibody produced in rabbit (*SIGMA; catalogue number: SAB4501422*); dilution: 1:500 Anti-Calnexin antibody (*Abcam*; *catalogue number: ab22595*); dilution: 1:1000. Anti-Rabbit secondary antibody (*Dako*); dilution: 1:2000.

## Additional Information

**How to cite this article:** Mosca, S. *et al*. Human *NDE1* splicing and mammalian brain development. *Sci. Rep.* **7**, 43504; doi: 10.1038/srep43504 (2017).

**Publisher's note:** Springer Nature remains neutral with regard to jurisdictional claims in published maps and institutional affiliations.

## Supplementary Material

Supplementary Figure 1
