# Supplementary material for: Human NDE1 splicing and mammalian brain development
Source: Sci Rep. 2017 Mar 7;7:43504. doi: 10.1038/srep43504 (PMC5339911; doi:10.1038/srep43504)
Supplement: Supplementary Figure 1 [file srep43504-s1.pdf]

Supplementary Info for:

Human NDE1 splicing and mammalian brain development.

S. Mosca, M. Raponi, A. Menegello, E. Buratti, C.G. Woods and D. Baralle

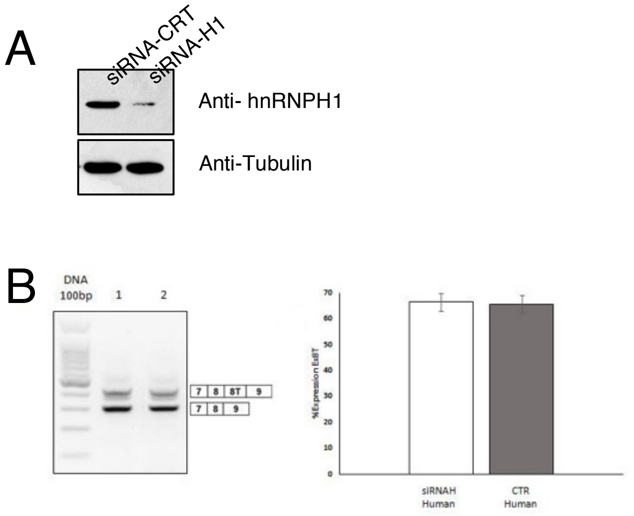

Supplementary Figure 1

A) The upper Western blot shows the efficiency of hnRNP H knockdown in SK-N-SH cells following treatment with specific siRNA against this protein (siRNA-H1) or luciferase siRNA control (siRNA-CRT). The lower blot shows a Western blot Anti-tubulin as a loading control.

B) The panel on the left shows the NDE1 splicing pattern in SK-N-SH cells transfected with 1) human NDE1 minigene and siRNA for hnRNP H; 2) human NDE1 minigene and luciferase siRNA control. The panel on the right shows a chart of band intensity percentage and skipping of exon 8T, relative to panel A.
